# Penile-scrotal erythrodysesthesia among rectal cancer patients receiving fluoropyrimidine-based chemoradiation: a case report series

**DOI:** 10.1007/s00384-024-04647-2

**Published:** 2024-05-23

**Authors:** Angela Adames, Diana Roth O’Brien, Alison R. Kelly, Leonard B. Saltz, Julio Garcia-Aguilar, Melissa Zinovoy, Vonetta Williams, Abraham Wu, Marsha Reyngold, Carla Hajj, Christopher Crane, Andrea Cercek, J. Joshua Smith, Alina Markova, John Cuaron, Patrick McCann, Paul B. Romesser

**Affiliations:** 1 Department of Nursing, Memorial Sloan Kettering Cancer Center, New York, NY USA; 2 Department of Radiation Oncology, Memorial Sloan Kettering Cancer Center, New York, NY USA; 3 Department of Medical Physics, Memorial Sloan Kettering Cancer Center, New York, NY USA; 4 Department of Medicine, Gastrointestinal Cancer Service, Memorial Sloan Kettering Cancer Center, New York, NY USA; 5 Department of Surgery, Colorectal Cancer Service, Memorial Sloan Kettering Cancer Center, New York, NY USA; 6 Department of Medicine, Dermatology Service, Memorial Sloan Kettering Cancer Center, New York, NY USA; 7 Department of Medicine, Early Drug Development, Memorial Sloan Kettering Cancer Center, New York, NY USA

**Keywords:** Scrotal erythrodysesthesia, Penile erythrodysesthesia, Toxic erythema of chemotherapy, Capecitabine, Fluorouracil, Neoadjuvant chemoradiation, Rectal cancer, Case report

## Abstract

**Background:**

Palmar-plantar erythrodysesthesia (PPE) is a slowly developing cutaneous reaction commonly experienced by patients treated with fluoropyrimidines. While erythrodysesthesia normally presents in a palmar-plantar distribution, it can also present with genital involvement, but this presentation is likely underreported and incorrectly attributed to an acute reaction from radiation therapy. This article aims to define erythrodysesthesia of the penis and scrotum as a rare but significant side effect of capecitabine.

**Case presentation:**

We identified five cases of moderate to severe penis and scrotal erythrodysesthesia over a 2-year period at a large tertiary cancer center, representing an estimated incidence of 3.6% among male patients with rectal cancer who were treated with fluoropyrimidine-based chemoradiation within our institution.

**Conclusions:**

Improved understanding of erythrodysesthesia involving the penis and scrotum can facilitate early identification and treatment of symptoms, and possibly prevent the discontinuation or delay of cancer treatment in patients treated with capecitabine and similar drugs. These clinical advances would improve and prolong patient quality of life during cancer treatment and prevent complications that result in hospitalization.

## Background

Neoadjuvant fluoropyrimidine-based chemoradiation is a standard of care treatment for patients with locally advanced rectal cancer [1–3]. Concurrent fluoropyrimidine therapy is preferentially administered as oral capecitabine, although continuous infusion 5-fluorouracil (5-FU) is an acceptable alternative [1, 4, 5]. One common adverse reaction associated with fluoropyrimidine is hand-foot syndrome (HFS), also known as palmar-plantar erythrodysesthesia (PPE). However, it should be noted that toxic erythema of chemotherapy (TEC) has become a more general terminology also utilized in the literature to characterize erythrodysesthesia and a variety of other cutaneous reactions triggered by chemotherapeutic agents. Up to 50% of patients receiving capecitabine report signs and symptoms of PPE [6, 7]. PPE is characterized by erythema, swelling, desquamation, ulceration, tingling, and pain that typically presents in a palmer and/or plantar distribution [6]. The rate and severity of PPE can depend on specific chemotherapy combinations, dose, schedule, and even cancer diagnosis [7].

Over a 2-year period at a tertiary cancer center, we retrospectively identified five cases of moderate to severe erythrodysesthesia involving the penis and/or scrotum in male patients with rectal cancer who were treated with neoadjuvant fluoropyrimidine-based chemoradiation. Each patient exhibited a scrotal/penile cutaneous presentation of erythrodysesthesia, ranging from moderate erythema and irritation to severe painful desquamation and ulceration. While erythrodysesthesia with genital involvement is mostly reported in patients treated with oral fluoropyrimidine capecitabine, we also report a case of erythrodysesthesia with genital involvement in a patient treated with bolus 5-FU/leucovorin-based chemoradiation. A comprehensive review of radiation treatment plans confirmed minimal radiation dose (Table 1), well below the threshold for dermatitis, to the penis and scrotum in these patients. In the patients whose capecitabine was held, their symptoms started to improve a few days later despite continuing radiotherapy.

**Table 1:** Radiation dose to penis and scrotum

**Case No.**	**Penis** ***D*max (cGY)**	**Penis** ***D*mean (cGy)**	**Penis V20Gy (%)**	**Penis V30Gy (%)**	**Penis V40Gy (%)**	**Scrotum** ***D*max (cGy)**	**Scrotum** ***D*mean (cGy)**	**Scrotum V20Gy (%)**	**Scrotum V30Gy (%)**	**Scrotum V40Gy (%)**
1	2298	1382	29.6	0	0	1021	112	0	0	0
2	2239	1812	28.2	0	0	2127	706	1.3	0	0
3	4238	1983	46.9	7.04	0.31	1831	750	0	0	0
4	2591	1940	29.3	0	0	1968	432	0	0	0
5	1607	198	0.0	0	0	180	54	0	0	0

There is very limited literature describing erythrodysesthesia with genital involvement due to fluoropyrimidine-based chemotherapy regimens, with or without concurrent radiation administration, as summarized in Table 2. Consequently, the incidence of erythrodysesthesia of the penis and scrotum is unknown. Intriguingly, there are no reports, to our knowledge, of genital erythrodysesthesia among women receiving fluoropyrimidine-based therapies. Given the rarity of this presentation, its incidence is likely underreported or unrealized in most clinical settings due to an incorrect differential diagnosis (e.g., allergic reaction, radiation dermatitis, or cellulitis) [8–13]. Based on our experience, patients are also hesitant to report these symptoms until they are quite severe given their sensitive nature.

**Table 2:** Summary of literature

**Author**	**Year**	**Origin**	**Age/race/diagnosis (if known)**	**Drug regimen**	**Symptom onset**	**Drug discontinued**	**Drug dose reduced**
**Sapp and DeSimone** [8]	2007	USA	67-year-old man, White, T3N1M0 colon cancer	Capecitabine 1G BID (14 days on, and 14 days off)	Cycle 3	✔	_
			63-year-old man, White, T4N1M0 colon cancer and T1N0M0 gastric adenocarcinoma	Adjuvant capecitabine 1G BID (14 days on, and 14 days off) + Oxaliplatin	Cycle 1, day 12	✔	_
**Lee et al.** [9]	2009	Korea	65-year-old man, metastatic sigmoid cancer	Adjuvant 5FU + Oxaliplatin	Cycle 1, day 6	_	✔
**Fleta-Asin et al.** [10]	2011	Spain	84-year-old man, metastatic sigmoid cancer	Capecitabine	Symptoms endured for 4 out of 5 months of treatment	✔	_
			78-year-old man, metastatic colon cancer	Capecitabine	After first cycle	✔	✔
			73-year-old man, rectosigmoid carcinoma	Capecitabine with radiation therapy (5 weeks)	Last 4 weeks of radiation	_	_
**Ljubojevic Hadzavdic et al.** [11]	2017	Croatia	63-year-old man, metastatic caecum cancer	XELIRI (irinotecan and capecitabine) + bevacizumab	End of first cycle of chemotherapy	✔	_
**Hu et al.** [12]	2018	Canada	43-year-old man, White, low rectal adenocarcinoma	Standard dose capecitabine with radiation (28 fractions)	Week 5/fraction 23	✔	_
**Dricken et al.** [13]	2020	USA	87-year-old man, White, rectal adenocarcinoma	Neoadjuvant radiation with capecitabine	Fraction 11	Patient initially dose reduced then ultimately discontinued both capecitabine and radiation	_

This article has three aims: (1) describe the typical presentation of erythrodysesthesia with genital involvement, (2) estimate the incidence of genital erythrodysesthesia, and (3) demonstrate that the manifestation of erythrodysesthesia of the scrotum and penis secondary to fluoropyrimidine-based therapies (e.g., capecitabine and 5-FU) is more prevalent than reported in the literature.

## Case presentations

From January 2021 through December 2023, we identified five patients with rectal cancer who developed erythrodysesthesia with genital involvement during capecitabine- or fluorouracil-based chemoradiation (Table 3). All five patients were men, consistent with literature reports that are also exclusively limited to male patients. During this same time period, 224 patients with rectal cancer, including 140 men and 84 women, were treated with fluoropyrimidine-based chemoradiation. The estimated reported incidence of erythrodysesthesia was 2.2% among all patients, and 3.6% among male patients with rectal cancer undergoing neoadjuvant fluoropyrimidine-based chemoradiation.

**Table 3:** Summary of cases

Case report	Age/ethnicity	Rectal cancer stage	Radiation dose/fractionation	Symptom onset	Reaction severity	Chemotherapy held
1	52-year-old White male	cT3N0M0	54 Gy/30	Week 3	Grade 3	✔
2	46-year-old Asian male	cT3bN0M0	54 Gy/30	Week 5	Grade 2	
3	53-year-old White male	cT4N1M0	54 Gy/27	Week 4	Grade 3	✔
4	68-year-old White male	cT4bN+M0	54 Gy/30	Week 5	Grade 3	
5	67-year-old White male	T3bN+M0	54 Gy/30	Week 5	Grade 3	✔

### Case 1

**Fig. 1:**
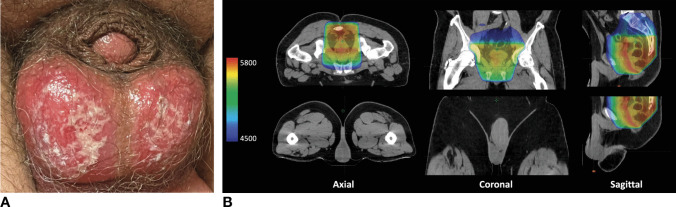
**A** Photograph of the scrotum of a 52-year-old man (patient 1) with cT3N0 rectal adenocarcinoma who underwent induction capecitabine-based chemoradiation and who developed painful, desquamation with purulent discharge to his scrotum during the fifth week of chemoradiation. **B** Representative images of his radiation treatment plan

Patient 1 is a 52-year-old White man with Mismatch Repair proficient (MMRp), cT3N0, and rectal adenocarcinoma that was 4 cm from the anal verge with possible sphincter involvement. He underwent induction chemoradiation to 54 Gy in 30 fractions with three-dimensional conformal radiotherapy (3D-CRT) and concomitant capecitabine 825 mg/m^2^ administered 5 days per week on days of radiotherapy. At the beginning of the fifth week of chemoradiation, he presented with painful, desquamation with purulent discharge to his scrotum (Fig. 1A). When questioned on symptom duration, the patient reported that he noticed mild scrotal irritation since the third week of chemoradiation that progressively worsened, but he failed to report it due to self-consciousness. His pain, which was exacerbated with walking, prevented him from performing the activities of daily living. Capecitabine was held for the sixth and final week of chemoradiation. Supportive skin care measures (including use of dermal wound cleanser, bacitracin, and dressings) were implemented daily in the clinic. His scrotal skin irritation began to improve a couple days after the cessation of capecitabine. Review of his radiation treatment plan confirmed no to minimal dose to the scrotum (Fig. 1B and Table 1). Despite his grade 3 erythrodysesthesia with genital involvement, he was able to complete all radiation treatments.

He then went on to receive consolidative chemotherapy with fluorouracil, irinotecan, and oxaliplatin (FOLFIRINOX) without reemergence of scrotal erythrodysesthesia.

### Case 2

Patient 2 is a 46-year-old Asian man with MMRp, cT3bN0, rectal adenocarcinoma that was 6–8 cm from the anal verge. He underwent induction chemoradiation to 54 Gy in 30 fractions with 3D-CRT and concomitant capecitabine 825 mg/m^2^ administered 5 days per week on days of radiotherapy. At the beginning of the fifth week of chemoradiation, he developed left testicular pressure and hyperpigmentation to the penis shaft and scrotum. During the sixth week, he reported worsening significant pain and edema of his penis and irritation of his scrotum. Review of his radiation treatment plan confirmed no to minimal dose to the penis and scrotum (Table 1). He was able to complete chemoradiation without interruption or capecitabine dose reduction despite developing grade 2 erythrodysesthesia with genital involvement. The patient was referred to dermatology, and his symptoms were managed conservatively with topical silver sulfadiazine, triamcinolone, aloe vera, and morphine gel applied to non-occlusive dressings. His symptoms resolved shortly after he completed chemoradiation—well in advance of improvement of his radiation side effects. He subsequently went on to receive consolidative capecitabine and oxaliplatin (CAPEOX) a month later. However, due to transient visual disturbance to his right eye after one cycle, oxaliplatin was omitted, and he completed chemotherapy with capecitabine alone. During this period, he experienced a re-emergence of his genital erythrodysesthesia, but this time, he also experienced erythrodysesthesia to his hands and soles of his feet, which began prior to his fourth cycle of capecitabine. His symptoms progressed from mild erythema and discomfort to eventual painful skin sloughing. This resulted in him having to hold capecitabine for 1 week to facilitate healing with topical clobetasol and celecoxib as needed, prior to completing his last six days of treatment. Following completion of treatment, these symptoms also eventually resolved.

### Case 3

**Fig. 2:**
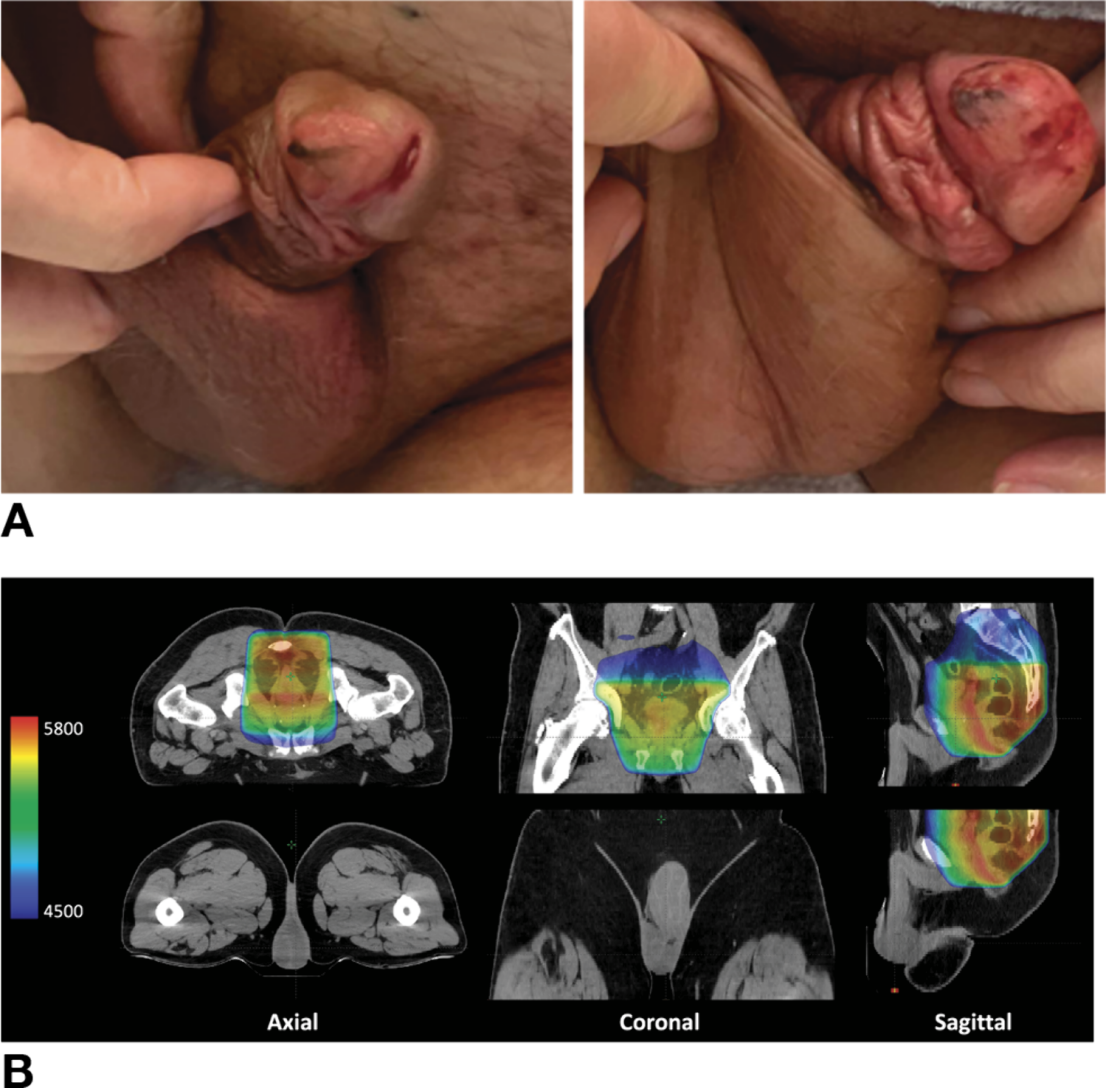
**A** Photograph of the penis of a 53-year-old man (patient 3) with cT4bN1, invasive adenocarcinoma who was treated with CAPEOX without complications and who developed painful penile ulceration during the fifth week of consolidative capecitabine-based chemoradiation. **B** Representative images of his radiation treatment plan

Patient 3 is a 53-year-old White man with MMRp, cT4bN1, moderately differentiated, invasive adenocarcinoma that was 2 cm from the anal verge. He was treated with induction CAPEOX without complications. He then underwent consolidative chemoradiation to 54 Gy in 27 fractions with volumetric modulated arc therapy (VMAT) and concomitant capecitabine 825 mg/m^2^ administered 5 days per week on days of radiotherapy. During his fourth week of chemoradiation, he reported mild erythema and irritation to the tip of his penis. By the fifth week of chemoradiation, he developed pain, tenderness, and skin sloughing with open blisters on his penile glans (Fig. 2A). Review of his radiation treatment plan noted a mean dose of 19.8 Gy to the penis and 7.5 Gy to the scrotum (Fig. 2B and Table 1). He was followed by dermatology for management of his penile skin erosion. The infectious work-up during this time was unremarkable. Dermatological management during this time included ketoconazole wash daily, in addition to topical ciclopirox and mupirocin twice daily to the ulcerated penile glans. While the last four doses of capecitabine were held given his grade 3 erythrodysesthesia with genital involvement, he was able to complete all radiation treatments.

### Case 4

**Fig. 3:**
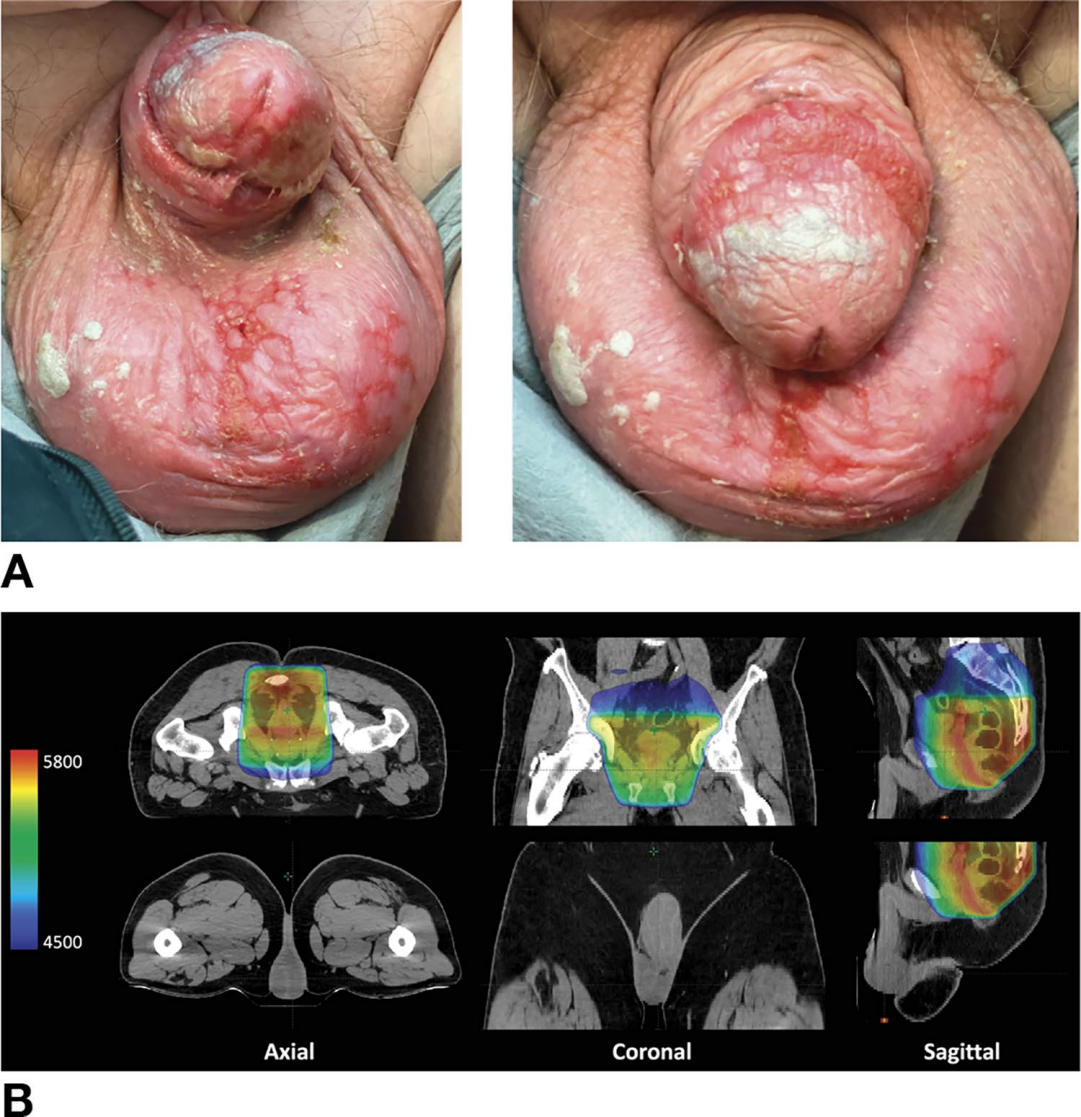
**A** Photograph of the penis and scrotum of a 68-year-old man (patient 4) with cT4bN+ , low rectal adenocarcinoma, who completed induction FLOX without dermatological complication, then underwent consolidative chemoradiation with bolus fluorouracil and presented with severe pain, desquamation, and fissuring to penis and scrotum in the sixth week of chemoradiation. **B** Representative images of his radiation treatment plan

Patient 4 is a 68-year-old White male with cT4bN+ rectal adenocarcinoma that was 3.5 cm from the anal verge. He completed eight cycles of induction fluorouracil, leucovorin, and oxaliplatin (FLOX) without dermatological complication. He then went on to receive consolidative chemoradiation to 54 Gy in 30 fractions with 3D-CRT and concomitant bolus 5-FU (425 mg/m^2^) and leucovorin (20 mg/m^2^). During his fifth week of chemoradiation, he developed erythema and irritation to the penile and scrotal skin to which topical silver sulfadiazine and an antifungal cream were prescribed for management. In the following week, this progressed to desquamation (including fissuring), bleeding, and severe pain to the penis (Fig. 3A). Review of his radiation treatment plan confirmed no to minimal dose to the scrotum (Fig. 3B and Table 1). Dermatology evaluation at the end of treatment confirmed that his symptoms were consistent with erythrodysesthesia due to 5-FU. The patient continued topical silver sulfadiazine but was also prescribed topical mometasone for dermatological management and oral tramadol for pain management. Despite developing grade 3 erythrodysesthesia with genital involvement, he had already completed all chemoradiation treatments. His symptoms resolved a few weeks after completing chemoradiation.

### Case 5

Patient 5 is a 67-year-old White male with synchronous cT3bN+ rectal adenocarcinoma and stage IV cecal adenocarcinoma metastatic to the liver. He completed induction FOLFOX (first-line) and fluorouracil, leucovorin, and irinotecan (FOLFIRI) with bevacizumab (second-line), followed by right hemicolectomy and liver wedge resection. He then went on to receive consolidative chemoradiation to 54 Gy in 30 fractions with 3D-CRT and concomitant capecitabine 825 mg/m^2^ administered 5 days per week on days of radiotherapy. During his fifth week of chemoradiation, he developed severe erythema, pain, and drainage along the scrotal skin. He was prescribed acetaminophen with codeine for pain management and advised by his local dermatologist to manage scrotal skin changes with topical hydrocortisone. However, he was eventually hospitalized with worsening scrotal skin pain and desquamation during his sixth week of chemoradiation. During his inpatient stay, necrotizing fasciitis and cellulitis were ruled out. Diagnostic work-up indicated he likely experienced a chemotherapy-induced skin reaction. The patient was followed by dermatology and infectious disease during his admission and was started on empiric intravenous and oral antibiotics, as well as topical silver sulfadiazine. While the last 3 days of capecitabine were held given his grade 3 erythrodysesthesia with genital involvement, he was able to complete all radiation treatments. His symptoms began to improve following discontinuation of capecitabine.

## Discussion and conclusions

The exact pathophysiology of PPE secondary to fluoropyrimidine administration is not fully understood. One theory suggests possible genetic polymorphism of specific genes—thymidylate synthase and dihydropyrimidine dehydrogenase (DPD)—may potentiate an individual’s intolerance to fluoropyrimidines like capecitabine and 5-FU and increase the potential for developing symptoms of PPE [14]. We do not routinely test for DPD deficiency at our center unless there are clinical symptoms suggestive of DPD deficiency. Another theory posits pathophysiological processes that facilitate the accumulation of fluoropyrimidine-based drugs to skin surfaces through eccrine glands, forming damaging free radicals in the upper epidermis that result in skin erythema or erosion [15]. Although the pathophysiology remains unclear, the association of PPE with capecitabine and 5-FU is well established [6, 7].

While PPE is a common adverse reaction attributed to fluoropyrimidine, rates of grade 3 PPE during chemotherapy and chemoradiation are fortunately low as dose reduction can help prevent grade 1–2 reactions from progressing to grade 3 reactions. In the National Surgical Adjuvant Breast and Bowel Project (NSABP) R-04 study, which demonstrated the equivalence of 5-FU and capecitabine-based chemoradiation, the rate of grade 3 PPE during chemoradiation was dose and schedule dependent [2]. When oral capecitabine 825 mg/m [2] was administered twice per day 7 days per week throughout the course of radiotherapy, the rate of grade 3 PPE was 3.5%. In comparison, when the daily dose of capecitabine remained the same but the frequency of capecitabine treatment was reduced from 7 days per week to 5 days per week, the rate of grade 3 PPE was 0.3% [2]. Similarly, in the Xeloda in Adjuvant Colon Cancer (X-ACT) study, which demonstrated the equivalence of oral capecitabine and bolus-fluorouracil plus leucovorin in the adjuvant setting for patients with stage III colon cancer, the rate of grade 3 PPE was 1.7% in patients treated with capecitabine at a dose of 1250 mg/m^2^ given twice daily on days 1 through 14 every 21 days [16]. Additionally, the International Duration Evaluation of Adjuvant Chemotherapy (IDEA) study found increased likelihood of developing grade 3 PPE in patients with stage III colon cancer treated with 6 months as compared to 3 months (2.6% vs. 0.7%) of adjuvant capecitabine [17]. As the rate of grade 3 PPE is dose and schedule dependent, early PPE identification and fluoropyrimidine dose modification can likely prevent a grade 1–2 reaction from progressing to a more severe grade 3 reaction.

This is the first report, to our knowledge, to calculate an estimated incidence for erythrodysesthesia with genital involvement (2.2% among all patients and 3.6% among male patients with rectal cancer treated with fluoropyrimidine-based chemoradiation). While our series is limited to patients undergoing fluoropyrimidine-based chemoradiation for rectal cancer, and patient identification for this case series was based on recall of cases occurring during January 2021 through December 2023, we believe that this incidence can reasonably be extrapolated to all patients treated with fluoropyrimidine-based systemic therapies. Our data suggests that erythrodysesthesia with genital involvement is more common than previously believed, and actual incidence may be higher due to underreporting or lack of clinical appreciation of its occurrence. It is important to consider erythrodysesthesia with genital involvement in male patients treated with fluoropyrimidines to allow early identification and intervention. Early intervention may mitigate progression to grade 3 toxicity, limit discontinuation or interruption of cancer treatment, improve and prolong patient quality of life, and prevent hospitalization due to complications related to infection, pain, and ulceration.

While the Common Terminology Criteria for Adverse Events (CTCAE) version 5 summarized in Table 4 specifically includes PPE, there is no formal grading criteria for erythrodysesthesia involving the penis and/or scrotum [18]. Therefore, the consideration of a more inclusive grading system that describes scrotal and penile erythrodysesthesia would further assist clinicians to identify genital erythrodysesthesia early on in therapy (e.g., at a grade 1 presentation). Indeed, in this case series, we extrapolated the CTCAE for PPE to systematically grade erythrodysesthesia with genital involvement.

**Table 4:** Palmar-plantar erythrodysesthesia toxicity grading via the Common Terminology Criteria for Adverse Events version 5

Palmar-plantar erythrodysesthesia syndrome	
Grade 1	Minimal skin changes or dermatitis (e.g., erythema, edema, or hyperkeratosis) without pain
Grade 2	Skin changes (e.g., peeling, blisters, bleeding, fissures, edema, or hyperkeratosis) with pain; limiting instrumental activities of daily living.
Grade 3	Severe skin changes (e.g., peeling, blisters, bleeding, fissures, edema, or hyperkeratosis) with pain; limiting self-care or activities of daily living.

Finally, with an increased awareness and identification of genital erythrodysesthesia, management goals should include a possible dose reduction or discontinuation of the specific fluoropyrimidine drug in conjunction with supportive skin and pain care measures in order to prevent progression of symptoms (i.e., to grade 2 or 3) and prevent interruption of therapy. Though there is no established standard to manage symptoms of PPE, literature cites drug interruption or dose reduction as the mainstay of treatment for TEC, based on the severity of reaction. Alternative supportive interventions may also include high potency topical corticosteroids, wound care for ulcerations, analgesics for pain management (e.g., opioids or gabapentin), and emollients and topical keratolytics (e.g., urea or salicylic acid) for area of keratosis [19, 20].

## Data Availability

No datasets were generated or analyzed during the current study.
